# Functional Characterization of Cryptococcal Genes: Then and Now

**DOI:** 10.3389/fmicb.2018.02263

**Published:** 2018-09-20

**Authors:** Lukas M. du Plooy, Olihile M. Sebolai, Carolina H. Pohl, Jacobus Albertyn

**Affiliations:** Pathogenic Yeast Research Group, Department of Microbial, Biochemical and Food Biotechnology, University of the Free State, Bloemfontein, South Africa

**Keywords:** biolistic transformation, CRISPR-Cas9, *Cryptococcus*, electroporation, gene targeting

## Abstract

Site-directed mutagenesis enables researchers to switch a gene of interest off for functional characterization of the gene. In the pathogenic yeasts, *Cryptococcus neoformans* and sister species *C. deneoformans*, this is almost exclusively achieved by introducing DNA into cells through either biolistic transformation or electroporation. The targeted gene is then disrupted by homologous recombination (HR) between the gene and the transforming DNA. Both techniques have downsides; biolistic transformation equipment is very expensive, limiting the use thereof to well-resourced laboratories, and HR occurs at extremely low frequencies in electroporated cryptococcal cells, making this method unappealing for gene targeting when not making use of additional modifications or methods to enhance HR in these cells. One approach to increase the frequency of HR in electroporated cryptococcal cells have recently been described. In this approach, CRISPR-Cas9 technology is utilized to form a double strand break in the targeted gene where after the occurrence of HR seems to be higher. The less expensive electroporation technique can therefore be used to deliver the CRISPR-Cas9 components into cells to disrupt a gene of interest, but only if the CRISPR components can be maintained for long enough in cells to enable their expression. Maintenance of episomal DNA occurs readily in *C. deneoformans*, but only under certain conditions in *C. neoformans*. In addition, CRISPR-Cas9 allows for gene complementation in order to fulfill Falkow’s molecular Koch’s postulates and adds other novel methods for studying genes as well, such as the addition of a fluorophore to an inactive Cas9 enzyme to highlight the location of a gene in a chromosome. These developments add less expensive alternatives to current methods, which could lead to more research on this yeast in developing countries where cryptococcal infections are more prevalent and researchers have access to more clinical isolates.

## Introduction

Site-directed mutagenesis is an essential tool for the functional characterization of genes and is therefore also used to identify virulence genes in pathogens. It involves disrupting or altering a gene of interest by exploiting homologous recombination (HR), the double strand break (DSB) repair machinery of cells, where the separated ends are joined by recombination with a homologous strand (Aylon and Kupiec, 2004). For gene disruption, a synthetic oligonucleotide, often referred to as donor DNA, is introduced into a cell which is then incorporated into the gene through HR (Kuwayama et al., 2008; Carrigan et al., 2011). Depending on the donor DNA utilized, disruption can be obtained through a frameshift mutation where an inactive or altered peptide is produced, or a deletion can be obtained with the insertion of a long stretch of bases, such as a reporter gene, resulting in the production of no protein at all. One can thereafter determine the change in the phenotype and ultimately deduce the function of the gene product. Before HR can take place, the donor DNA needs to be delivered into cells, which is often a more laborious process for cells with a thick cell wall or capsule, such as plant and fungal cells. This is also true for the yeasts *Cryptococcus neoformans* and *C. deneoformans*, the causative agents of cryptococcosis in immunocompromised patients (Goins et al., 2006).

Cryptococcosis is an infection of the pulmonary system of humans and other mammals and if untreated, the disease could progress to cause an often deadly inflammatory condition of the brain and spine (Nielsen et al., 2007; Djordjevic, 2010). *Cryptococcus neoformans* was first isolated between 1894 and 1895 by Busse and Buschke from a lesion of a woman’s tibia (Barnett, 2010; Espinel-Ingroff and Kidd, 2015). It was not yet possible to study the molecular mechanisms behind the pathogenesis of this yeast for close to century after this discovery, despite knowledge of its virulence.

All of the pathogenic *Cryptococcus* species were initially classified as varieties of a single species, *C. neoformans* (Espinel-Ingroff and Kidd, 2015). In 2002, molecular characterization and other work led to the recognition of *C. neoformans* var. *gattii* as the distinct species *C. gattii* (Kwon-Chung et al., 2002; Kwon-Chung and Varma, 2006). Another reclassification was proposed by Hagen et al. (2015) following a debate between proponents of the old classification and a revised classification of these yeasts. *Cryptococcus neoformans* var. *neoformans* (also commonly referred to as serotype D) was renamed to *C. deneoformans* and *C. neoformans* var. *grubii* (serotype A) retained the name *C. neoformans*. The five genotypes of *C. gattii* were also raised to species level, yielding a total of seven species in the new *C. neoformans*/*gattii* species complex.

In the 1980s, virulence studies on *C. neoformans* and *C. deneoformans* were done using non-specific mutagenesis, most notably by the Kwon-Chung group. Mutants lacking the most apparent virulence traits (i.e., capsule and melanin formation as well as growth at 37°C) were generated with UV irradiation and subsequent cloning (Kwon-Chung et al., 1982; Rhodes et al., 1982; Kwon-Chung and Rhodes, 1986). However, nothing about the molecular mechanisms behind these mutants were known, and site-directed mutagenesis only became possible in *Cryptococcus* species when Edman and Kwon-Chung (1990) adopted an electroporation protocol optimized for *Saccharomyces cerevisiae* to introduce foreign DNA into the cells. With this technique, cell membranes are made more permeable by exposure to an electrical impulse to facilitate the transport of particles, such as DNA, across the membranes (Neumann et al., 1982). Electroporation was first developed by Neumann et al. (1982) for the transfection of mouse lyoma cells and could deliver DNA into cryptococcal cells more readily than chemical transformation methods used for *S. cerevisiae*, such as the lithium acetate method described by Ito et al. (1983). In fact, to our knowledge the only reference in literature made to a chemical transformation method of cryptococcal cells was by Ou et al. (2011), who used a “lithium acetate yeast transduction kit” to introduce DNA into *C. neoformans*. Chemical transformation methods are very inefficient in these yeasts, due to the thick capsule and cell wall that must be crossed by the transforming DNA (Srikanta et al., 2014).

A second technique, biolistic transformation, involves ballistically transforming cells with DNA-coated metal microparticles (Klein et al., 1987; Toffaletti et al., 1993). Biolistic transformation was originally developed by Klein et al. (1987) to transfect plant cells in hopes of circumventing some of the limitations faced with delivering DNA into these cells, such as getting enough DNA through the thick cell wall. Since fungi are also covered with a thick cell wall, this technique is also frequently used to deliver DNA into fungal cells, such as *Trichoderma harzianum* and *Gliocladium virens* (Lorito et al., 1993). As was the case with electroporation, a biolistic transformation protocol was borrowed from *S. cerevisiae* and adapted for *C. neoformans* by Toffaletti et al. (1993). This method yields higher transformation and HR efficiencies than electroporation and have since been established as the method of choice by many *Cryptococcus* researchers (Lin et al., 2014; Srikanta et al., 2014). Other attempts at transforming these yeasts include protoplasting and *Agrobacterium*-mediated transformation (AMT) (McClelland et al., 2005; Lin et al., 2014; Srikanta et al., 2014). *Agrobacterium tumefaciens* is a gram-negative soil bacterium that is capable of transferring a Ti plasmid vector carrying the T-DNA (transfer DNA) into plant and fungal cells for integration into a host chromosome (McClelland et al., 2005; Srikanta et al., 2014). Both techniques are very ineffective in achieving site-directed mutagenesis and is therefore not used for gene characterization (Lin et al., 2014). *Agrobacterium*-mediated transformation is less time consuming than preparing protoplasts (McClelland et al., 2005) and does yield high transformation efficiency and stable transformants, but does not mediate HR (Srikanta et al., 2014).

## RNA-Mediated Gene Silencing

An alternative to the DNA targeting techniques described above is to target the transcription product, messenger RNA (mRNA), instead to ultimately elucidate the role of the relevant gene. Napoli et al. (1990) discovered that supplementing petunia plants (*Petunia* spp.) with an additional copy of the chalcone synthase (CHS) gene, one of the genes responsible for the violet pigment in petunia flowers, unexpectedly yielded white flowers instead. They concluded that the transferred gene somehow caused both the endogenous and transferred gene to be suppressed. Further studies revealed that introducing a double-stranded RNA (dsRNA) sequence homologous to a sequence in a cell results in silencing of the gene (Fire et al., 1998). It was initially thought that silencing occurred when the antisense strand bound to complementary mRNA, marking it for degradation. Two independent groups (Hammond et al., 2000; Zamore et al., 2000) showed that this was not entirely the case; an enzyme processes dsRNA into small interfering RNA (siRNA) of about 21–23 nucleotides. The enzyme, known as Dicer, was later identified by Bernstein et al. (2001). Dicer, a member of the RNase III family, therefore digests dsRNA into mature siRNAs. Further work showed that these short siRNA molecules then enter an assembly pathway with effector assemblies known as RNA-induced silencing complexes (RISCs), which facilitates duplex unwinding by a protein known as argonaute protein (Carthew and Sontheimer, 2009). This RNA-protein complex is then responsible for the sequence specific cleavage of mRNA using the siRNA as guide (Skowyra and Doering, 2012).

This cellular process can therefore be exploited to silence the expression of targeted genes by introducing a dsRNA molecule homologous to the mRNA of a targeted gene into cells. This dsRNA molecule can be synthesized *in vivo* or *in vitro* and then introduced into cells with electroporation (Liu et al., 2002). Gorlach and co-workers discovered in 2002 that RNA-mediated gene silencing functions in both *C. neoformans* and *C. deneoformans* (Skowyra and Doering, 2012). They successfully suppressed expression of the calcineurin A (*CNA1*) gene in *C. deneoformans* and laccase (*LAC1*) gene in *C. neoformans.* Another group, Liu et al. (2002), suppressed *CAP59*, a gene involved in capsule synthesis and *ADE2*, a gene in the adenine biosynthetic pathway in *C. deneoformans*. RNA interference (RNAi) have several advantages over conventional gene disruption techniques relying on HR (Skowyra and Doering, 2012). For instance, the *in vivo* synthesis of the dsRNA can be driven by various promoters, such as inducible promoters which adds more control over when a gene is targeted. However, the effect of a gene is not entirely eliminated with RNAi as is the case with gene deletion via HR; genes are “knocked down” instead of “knocked out” (Skowyra and Doering, 2012). The level of expression after RNA-mediated gene silencing depends on a number of factors, including the kinetics and stability of gene expression and the efficiency of the interference. This property can, however, be exploited to determine the function of essential genes, which would not be possible with gene deletion techniques.

## Electroporation vs. Biolistic Transformation

Both electroporation and biolistic transformation are frequently used molecular techniques when studying *C. neoformans* and *C. deneoformans* (Lin et al., 2014; Wang et al., 2016). However, gene targeting only became widespread when biolistic transformation was established (Lin et al., 2014; Srikanta et al., 2014). This technique has since been established as the preferred method for transforming these yeasts and significant progress have been made in identifying the genes behind some of the more prominent cryptococcal virulence factors using this method, such as the identification of genes that play a role in growth at 37°C. In fact, the first virulence gene replaced through biolistic transformation was the *n*-myristyl transferase (*NMT1*) gene which resulted in an avirulent temperature sensitive myristic acid auxotroph (Lodge et al., 1994; Perfect, 2006). Between 17.5 and 100% of transformants obtained with biolistic transformation are stable, while the majority of transformants obtained with electroporation are unstable; especially when auxotrophic markers are used (Lin et al., 2014). Electroporated cells transformed with these markers tend to lose the plasmid vectors after only a few generations, even when maintained on selective media (Varma and Kwon-Chung, 1992). This indicates that these vectors are maintained episomally instead of being integrated into the genome, either ectopically or through HR. Drug resistance markers generally yield better results with electroporation and Varma and Kwon-Chung (2000) achieved a 100% stability by using a cycloheximide resistance marker for selection. The transformation efficiency was low, however, and it was proposed that genomic integration was a requirement in this case for the cells to survive selection.

Homologous recombination did not necessarily occur in all stable transformants, which is required to obtain mutants lacking the targeted gene product. Biolistic transformation yields a HR frequency of between 2 and 50% in *C. neoformans* (Davidson et al., 2000). It has been shown that HR varies depending on the gene and strain, making frequencies between 1 and 10% more typical (Lin et al., 2014). In congenic *C. deneoformans* strains biolistic transformation yields a HR frequency of ∼1–4% (Davidson et al., 2000). In contrast, the HR frequency obtained with electroporation varies between 0.00001 and 0.001% for *C. deneoformans* (Davidson et al., 2000). Electroporation alone in *C. neoformans* is very inefficient and is therefore generally not applied to this yeast without help from other techniques to increase the frequency of HR. Toffaletti et al. (1993) obtained no stable transformants using only electroporation. However, Lin et al. (2014) had some success, where HR occurred in 2 out of 140 stable transformants when a G418 (geneticin) resistance marker was used; whereas no HR occurred in a total of 15 stable transformants when a nourseothricin resistance marker was used in genetically identical cells. It is therefore clear that the type of selection marker plays a role in the success of electroporation. The less favorable outcome of electroporation has been attributed to the inability of this technique to deliver DNA to the nucleus (Davidson et al., 2000). This does not, however, explain how electroporated stable transformants can grow on selective media without being able to migrate the DNA to the nucleus in order to express the selection marker.

Various modifications were made to plasmid vectors or cryptococcal cells to enhance stability or the frequency of HR, especially when using electroporation as a transformation method. The presence of an autonomously replicating sequence (ARS), obtained by the interaction of transforming DNA with the host genome, has enhanced the maintenance of plasmid vectors as extrachromosomal plasmids (Varma and Kwon-Chung, 1992, 1998). Varma and Kwon-Chung (1998) isolated such an ARS-like sequence, referred to as a “STAB” element, from a minichromosome obtained from stable electroporated cryptococcal cells and added it to otherwise unstable plasmids to enhance stability. It was later shown that this sequence originated from *Escherichia coli* and had no effect on the stability of transformants (Hull and Heitman, 2002). Telomeric repeats added to the end of a linearized vector did, however, increase stability of electroporated transformants (Edman, 1992).

More recent improvements include Ku mutants and the use of split-markers for selection which both lead to a higher HR frequency. The Ku mutant approach by Goins et al. (2006) involves deleting the genes encoding the Ku70–Ku80 heterodimer, which play a role in non-homologous end-joining (NHEJ), another cellular process responsible for repairing DSBs. This DNA repair process seems to be the preferred process in *C. neoformans* and *C. deneoformans*, explaining the low HR frequencies seen in these pathogens, even when biolistic transformation is employed (Arras and Fraser, 2016). The inability to repair DSBs with NHEJ increases the frequency of HR to almost 100%, although Ku mutants show altered virulence in mice and expression of the *KU80* gene is upregulated during human infection, making Ku mutants unsuitable for virulence studies (Arras et al., 2016). However, the use of chemical inhibitors of the NHEJ pathway could circumvent these effects on virulence. Arras and Fraser (2016) tested eight inhibitors of mammalian NHEJ and found that four influenced the rate of HR for multiple targeted genes in *Cryptococcus neoformans*. N-(6-aminohexyl)-5-chloro-1-naphthalenesulfonamide (W7), an inhibitor of the production of the Ku cofactor inositol hexakisphosphate, performed the best and is relatively inexpensive. In the split-marker approach, the selection marker is split into two fragments, requiring recombination to function (Fu et al., 2006). The likelihood of two additional recombination events occurring in the targeted gene is thereafter higher and increases the frequency of HR up to eight times when *URA5* is used as a selection marker.

Biolistic transformation is clearly the better technique, especially in *C. neoformans*, while electroporation can still be used to transform wild type C. *deneoformans* if HR is not a requirement. Inhibitors of NHEJ can be used to achieve HR with electroporation of cryptococcal cells, although this approach have thus far not been widely adopted. *Cryptococcus neoformans* is responsible for more than 90% of all cryptococcal infections worldwide and has the highest growth rate at 37°C of all *Cryptococcus* species, making this species the most virulent (Litvintseva et al., 2011; Hagen et al., 2015). It therefore makes sense to do virulence studies on *C. neoformans* species instead of *C. deneoformans*. Biolistic transformation is, however, generally more expensive than electroporation. The Biolistic^®^ PDS-1000/He Particle Delivery System sold by Bio-Rad Laboratories, Inc. was the first commercially available system and has been established as the most frequently used system by 1998 (Kikkert, 1993; Hagio, 1998). Most of the molecular work on *Cryptococcus* species. involving biolistic transformation most frequently rely on the Bio-Rad system since the first protocol for biolistic transformation of cryptococcal cells made use of helium for particle delivery, the approach this system was based on (Toffaletti et al., 1993). The Bio-Rad Biolistic^®^ PDS-1000/He Particle Delivery System costs US$ 33,000 compared to US$ 8,245 (listed prices as on June 2018) for a Gene Pulser Xcell^TM^ Electroporation System also sold by Bio-Rad Laboratories, Inc. This high price is furthermore accompanied by expensive consumables, such as gold beads, macrocarriers, stopping screens, and rupture disks (Lin et al., 2014), whereas the only additional equipment required for electroporation is reusable electroporation cuvettes. This restricts the use of the biolistic method to well-resourced laboratories. Slightly cheaper apparatus could, however, be obtained, such as hand-held gene guns and bench-top particle delivery systems from other manufacturers under-represented in literature as well as components for do-it-yourself (DIY) particle delivery systems (Hanson et al., 2016), although the latter option is accompanied by trade-offs involving safety, control over bombardment power, and consistency from transformation to transformation.

## The CRISPR-Cas9 Revolution

The development of a CRISPR-Cas9 system for gene targeting brought about a major breakthrough in genetic engineering, allowing researchers to target genes more accurately than ever before (McCarthy and Walsh, 2017). CRISPR, or Clustered Regularly Interspaced Short Palindromic Repeats, and the associated genes (*CAS*-genes) is a set of genes used by prokaryotes to protect themselves against invading genetic elements, such as viruses and plasmids (Van der Oost et al., 2009). This is found in over 88% of all archaeal genomes and 30% of bacterial genomes. Ishino et al. (1987) first noticed the CRISPR array in *E. coli* but paid little further attention to these repeats. The function only became a subject of research when Mojica et al. (1993) discovered similar repeats in *Haloferax mediterranei* and finally reported on their origin and possible function in 2005 (Mojica et al., 2005).

Further research revealed that foreign DNA are digested upon entering prokaryotic cells and integrated as “spacers” between two palindromic repeats in the CRISPR array by the Cas1 and Cas2 proteins (in most cases) in the acquisition phase (Levy et al., 2015; Makarova et al., 2015). Both the spacers and repeats are about 20–50 base pairs in length and new spacers are integrated next to a 100–500 base pair AT-rich region referred to as the leader sequence, which is believed to serve as a promoter for CRISPR transcription (Nuñez et al., 2014; Zhang and Ye, 2017). During the expression phase, the CRISPR array is transcribed and processed to mature CRISPR RNAs (crRNA) consisting of a spacer and one of the adjacent repeats (Arras et al., 2016). A single crRNA then associates with a Cas protein or protein complex and guides the effector complex to a complementary sequence on invading DNA, where the Cas nuclease creates a DSB during the interference phase (Zhang and Ye, 2017). The Cas protein or protein complex recognizes a ∼2–4 base pair protospacer adjacent motif (PAM) sequence on the invading DNA which is absent from the spacer to prevent digestion of the CRISPR array (Nuñez et al., 2014). The diverse CRISPR systems are classified into two classes: class 1, if a multi-subunit protein complex is involved in the interference phase and class 2, if a single protein is responsible for the interference phase (Zhang and Ye, 2017). These systems are further subdivided into types based on the signature proteins in the system.

The potential of this molecular immune system for gene targeting became apparent when it was discovered that the Cas nuclease was a programable restriction enzyme (Marraffini and Sontheimer, 2008). This notion was reinforced when Jinek et al. (2012) showed that Cas9 could cut DNA *in vitro* and that the enzyme can be programmed with custom-designed crRNAs. This group also showed that crRNA and trans-activating crRNA (tracrRNA – a short RNA molecule involved in pre-crRNA processing and binding to Cas9) can be joined into a single guide RNA (sgRNA or gRNA), simplifying the system for use by researchers. This was soon followed by the first *in vivo* use of CRISPR-Cas9 in eukaryotes by Cong et al. (2013), who used this technology to target genes in human and mouse cells. CRISPR-Cas9 was quickly adopted and modified by the research community for various roles. For instance, the nuclease activity of the Cas protein has been disrupted and bound to other proteins or molecules to study genes and non-coding regions (Barrangou and Doudna, 2016). Some roles performed thus far include transcriptional activation or transcriptional repression; imaging achieved by the addition of a fluorophore to the inactivated or “dead” Cas9 protein (dCas9) and epigenetic state alteration by bringing epigenetic repressors or activators to genes.

In 2016, two separate research groups applied CRISPR-Cas9 to *C. neoformans* and *C. deneoformans* research for the first time. Wang et al. (2016) constructed two cassettes containing the gDNA (guide DNA, to be transcribed to gRNA) and *CAS9* nuclease gene, respectively, and made use of electroporation to deliver the cassettes into *C. deneoformans* cells. A human codon optimized *CAS9* nuclease gene, fused to two nuclear localization signals, was employed and placed under the control of a *ACT1* promoter and tailed by a bGHpA terminator. The gDNA was placed under the control of a native U6 gene promoter and 6-T terminator for gRNA production. The cassettes were co-transformed into this yeast and the gRNA was designed to target the *ADE2* gene, creating an adenine auxotroph that forms pink colonies on plates with a low level of adenine. About 82–88% of transformants were pink and sequencing revealed various indel mutations most probably introduced through NHEJ. This group also showed that a single codon change in a targeted gene is possible when a donor DNA cassette is included for HR. Similarly, a hygromycin B resistance marker was introduced into a gene through HR. In the last two instances, the *CAS9* gene and gDNA were both on one vector, allowing co-transformation with the donor DNA.

**FIGURE 1:**
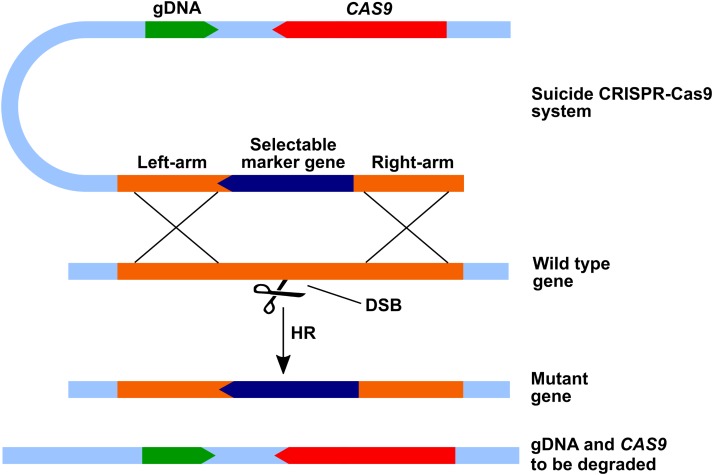
The “suicide” CRISPR-Cas9 system. The CRISPR components are carried on one vector together with a selectable marker flanked by sites homologous to the targeted gene. The Cas9-gRNA causes a double strand break (DSB) after which the selectable marker is introduced into the gene through homologous recombination (HR). The rest of the vector containing the CRISPR components is degraded (adapted from Wang et al., 2016).

The real significance of the paper by Wang et al. (2016) was the development of a “suicide” system that got rid of the CRISPR components after a gene has been targeted (**Figure 1**). In this system, the gDNA with or without the addition of *CAS9* are included on the vector with the insert flanked by sites homologous to the targeted gene. After HR, the section of the vector containing the CRISPR components are degraded. Success rates of almost 50% were obtained, even with large fragments containing both the gDNA and *CAS9* gene. Although, HR occurred much more frequently than was seen before, the expression of *CAS9* seemed to diminish the virulence of *C. neoformans* strain H99. This was in contrast with the findings of Arras et al. (2016), the second group to utilize CRISPR-Cas9 in these pathogens. These authors found that *CAS9* expression in *C. neoformans* H99 has no effect on growth, virulence factors or ability to cause disease in a murine inhalation model. The two-step system developed by this group first involved constructing a strain that expresses the *CAS9* gene after integration into a gene-free region of the genome. This is a useful approach which lessens the workload for targeting a series of genes, requiring only the addition of gDNA in subsequent studies. Arras et al. (2016) achieved this *CAS9* integration with biolistic transformation, which yields higher HR rates as was previously seen. Instead of using a RNA polymerase III promoter for gDNA transcription, the authors added two ribozyme genes to the ends of the gDNA. Upon transcription, the fragment undergoes self-cleavage liberating an unaltered gRNA molecule, as described by Gao and Zhao (2014). The advantage of this approach is that any promoter can be used for gDNA transcription. Similar to Wang et al. (2016), Arras et al. (2016) also targeted the *ADE2* gene as proof of concept, adding a successful two-step system to the one-step system of Wang et al. (2016).

## A New Hope for Electroporation

Two problems are often encountered when utilizing CRISPR-Cas9: overexpression of the *CAS9* gene that has been shown to be toxic in some fungi, including *S. cerevisiae* and *Schizosaccharomyces pombe*, and off-target mutations that accumulate over time (Wang et al., 2016). The “suicide” CRISPR-Cas9 system developed by Wang et al. (2016) for *C. deneoformans* solves these problems by getting rid of the CRISPR components after the DSB has been made. Before developing this system, they experimented with various methods to get rid of the CRISPR components after gene targeting, which included relying on the tendency of these yeasts to lose extrachromosomal DNA after a few generations, but found that the specific transformants tested remained stable. The “suicide” system also allows for restoration of the disrupted gene for the fulfillment of Falkow’s molecular Koch’s postulates (Falkow, 1988) and therefore seems like an elegant solution to the challenges faced previously with gene disruption in these yeasts by increasing the occurrence of HR and allowing the use of the less expensive electroporation method for transformation. In contrast, Arras et al. (2016) found that the *CAS9* gene must be integrated into the genome of *C. neoformans* H99 for CRISPR-Cas9 to work in this species. An increase in the frequency of HR was not seen when they co-transformed cells with the gDNA, *CAS9* gene and donor DNA on separate vectors without adding selective pressure to integrate or maintain the CRISPR components, indicating that *C. neoformans* is unable to stably maintain episomal constructs like *C. deneoformans*. Lin et al. (2014), however, obtained 140 stable *C. neoformans* H99 transformants out of 164 total transformants when a G418 resistance gene was used as a selection marker. A transiently expressed system, without *CAS9* genomic integration, could therefore also work in *C. neoformans* if a G418 marker provides selective pressure.

Fan and Lin (2018) recently showed that such a transient system, delivered via electroporation, works well in both *C. neoformans* and *C. deneoformans*. They referred to this system as a TRACE (transient CRISPR-Cas9 coupled with electroporation) system and targeted the *ADE2* gene to evaluate effectiveness. The two CRISPR-Cas9 components, *CAS9* gene and gDNA, were placed on separate vectors. The *CAS9* gene was placed under the control of a *GPD1* promoter and terminator while expression of the gDNA was driven by the U6 promoter and a 6-T terminator. A deletion construct containing a nourseothricin resistance gene flanked by arms homologous to the *ADE2* gene was constructed and delivered into cells with electroporation together with the CRISPR-Cas9 components. More than 90% of the *C. neoformans* transformants turned pink, while more than 50% of *C. deneoformans* strain JEC21 and more than 65% of *C. deneoformans* strain XL280 transformants turned pink. Stability assays revealed that most of the transformants retained the pink phenotype while losing the *CAS9* and gDNA vectors since these vectors did not include resistance genes for selection. This group further showed that the rate of gene disruption positively correlates with the dose of *CAS9* and gDNA vectors and that multiple closely related genes can be deleted with one transformation step if the short stretch of gDNA selected allows for targeting of these genes. As proof of concept, the mating type genes, *MFα1-4*, were deleted.

Another laboratory also recently exploited CRISPR-Cas9 technology to improve the practicality of electroporation for gene targeting in *Cryptococcus* species. Wang (2018) described two distinct methods to target *GIB2*, a highly conserved gene in *C. neoformans* and *C. deneoformans*, which encodes an atypical Gβ-like/RACK1 protein. The first technique is similar to the technique described by Fan and Lin (2018), where a transient plasmid carrying CRISPR-Cas9 genes is electroporated into cells for expression *in vivo*. In contrast to the system developed by Fan and Lin (2018), Wang (2018) placed both the *CAS9* gene and gDNA on a single plasmid for electroporation together with the donor DNA. The second technique relied on ribonucleoprotein-mediated CRISPR-Cas9 gene editing. Custom made crRNA for targeting the *GIB2* gene was mixed and incubated at 94°C with universal tracrRNA to facilitate annealing whereafter purified Cas9 protein was added before incubation to allow ribonucleoprotein complex formation. This complex was introduced into cryptococcal cells via electroporation together with donor DNA. Both techniques yielded *GIB2* mutants, although the DNA-based technique seemed to yield more transformants.

## Conclusion

Even though the biolistic transformation method contributed significantly to what is currently known about *C. neoformans* and *C. deneoformans*, low HR frequencies are still seen in biolistically transformed cells. Low transformation and HR frequencies are often seen in other fungi as well, including other pathogenic fungi. Fu et al. (2006) stated that a high frequency of gene disruption is an exception rather than the norm in fungal pathogens, which complicates functional characterization of genes. Not only is determining the function of genes important for basic research, but a deep understanding of the workings of virulence factors and the genes encoding them is required to elucidate the mechanism of action of potential drugs. The fundamental challenge to antifungal drug development is the conserved status of many biological processes between humans and fungi, which complicates clinical trial design (Roemer and Krysan, 2014). In addition to this challenge, the low transformation and HR frequency in pathogenic fungi has further slowed the development of novel antifungals in recent times (McCarthy and Walsh, 2017).

The Bio-Rad Biolistic^®^ PDS-1000/He Particle Delivery System is by no means a common sight in a microbiology laboratory and the high cost involved in acquiring such a system and the accompanying consumables have thus far restricted molecular research on *C. neoformans* and *C. deneoformans* to well-resourced research centers. Ironically, the high cost excludes many research laboratories in developing countries, which is also usually the worst affected areas due to immune deficiency caused by the AIDS pandemic. By enabling the use of electroporation, CRISPR-Cas9 technology could therefore bring *Cryptococcus* research right into the midst of the underdeveloped affected areas where researchers have access to more clinical isolates and could supplement the technology currently available to accelerate the discovery of novel drug targets.

Current treatment options include three old and off-patent drugs, amphotericin B (and its liposomal derivatives), 5-fluorocytosine and fluconazole (Perfect and Bicanic, 2015). Due to the high cost and inadequate supply chains, 5-fluorocytosine and amphotericin B drugs frequently do not reach patients in the most affected areas, such as sub-Saharan Africa (Perfect, 2013; Perfect and Bicanic, 2015). This is further made worse by difficulties with monitoring and managing the life-threatening adverse effects of amphotericin B (Perfect and Bicanic, 2015). Fluconazole is, however, donated and distributed by the Pfizer, Inc. Diflucan Partnership Program, which started as an agreement between Pfizer, Inc. and the South African Department of Health and currently provides support to many developing countries across the globe (Wertheimer et al., 2004). Fluconazole is, however, a fungistatic drug and lifelong maintenance therapy is therefore required (Vensel, 2002). This suppressive therapy frequently leads to relapse of this disease in patients in developing countries due to interactions with other drugs, poor compliance with treatment, malabsorption or the development of drug resistance with long term use (Armengou et al., 1996). There is therefore a need for combination therapy to reduce the chance of anti-fungal resistance and to shorten the treatment time (Vanden et al., 1998; Perfect, 2013; Perfect and Bicanic, 2015). No newly developed therapies reached patients in more than 25 years (Perfect, 2017). Treatments currently in the pipeline include APX001; a first-in-class compound that hinders the attachment of adhesion proteins to the outer cell wall, T-2307; an allylamine compound that inhibits the mitochondrial membrane potential and AR-12; a broad-spectrum antifungal for which the specific method of action is still unknown, but probably functions by blocking acetyl-CoA synthetase 1 and by downregulating host chaperone proteins (Perfect, 2017). The development of safer and cheaper treatment options could contribute tremendously to the fight against cryptococcosis, enabled by the new molecular techniques such as the CRISPR-Cas9 gene-targeting tool. Such techniques could prove to be invaluable in studies on the mechanism of action of potential antifungals. CRISPR-Cas9 is therefore not only a valuable healthcare tool that could directly combat human genomic diseases, but is also a valuable search tool in the pursuit of new drug targets in pathogens.

## Author Contributions

All authors are in agreement with the content of the manuscript. LP conducted the literature study and wrote the draft manuscript. JA, OS, and CP provided inputs, revised, and edited the manuscript.

## Conflict of Interest Statement

The authors declare that the research was conducted in the absence of any commercial or financial relationships that could be construed as a potential conflict of interest.
